# Medical and Physical Intelligence

**Published:** 1822-07

**Authors:** 


### Medical and Physical Intelligence.

### 1. *Royal Society of London.*

THE following papers connected with the medical sciences have been recently read:

*April* 25.—On the Mechanism of the Spine, by Mr. EARLE.

*May* 2.—On the Nerves which associate the Muscles of the Chest in the Actions of Breathing, Speaking, and Expression; by CHARLES BELL, Esq.

*May* 9.—Experiments and observations on the Newry Pitch-Stone, and on the artificial Formation of Pumice; by the Right Hon. J. KNOX.

*May* 16.—On the Changes which the Egg undergoes during Incubation; by Sir E. HOME, Bart.

2. *Re-union of the Osseous Disc, separated by the Operation of Trepan.*

Dr. WALTHER, Professor of Medicine and Surgery at Bonn, relates the following experiments, showing the re-union of the osseous disc, after it has been separated by the operation of trepan.

The left parietal of a dog, with a very small portion of the frontal bone, was laid bare by a crucial incision. A small trephine was applied to the parietal bone, close to the sagittal suture. The teeth of the instrument, after perforating the bone, had torn the dura mater, where it forms the ensiform sinus, and had opened the latter. Having removed the disc, the blood escaped with rapidity; but the hemorrhage was quickly stopped by means of lint. During this interval the bone, separated from the cranium and all the soft parts, remained upon the table. The periosteum was also removed from it. Perceiving no hemorrhage to follow the removal of the charpie which had been applied to the wound of the sinus, the osseous disc was replaced in the situation from which it had been taken. It did not entirely fill the hole in the parietal bone. The flaps formed by the scalp were placed over it, and their margins kept in contact by means of a suture.

The animal suffered little during the operation. On the second day he had slight fever, but on the third regained his appetite. The dog lived twelve months afterwards. After his death, the portion of bone formerly removed was found to be united to the margin of the opening, so as to render it difficult to discover its limits: so closely did the callus resemble the rest of the osseous substance, that they could not be distinguished.

The following case, which occurred in the human subject, is still more important:

A man was wounded on the head by a stone. The symptoms of concussion were moderate. He was bled on the second day after the accident; and on the sixth he felt himself so far recovered as to pursue his occupation. Pain of the head soon became so violent as to render him incapable of work. He was bled from the arms and feet, had cold lotions and ice to the head, vesicatories and setons to the nape of the neck, with purgatives and emetics, without relief. At length the pains in the head became intense: no other symptom, indicating lesion of the brain, was present. This determined individual begged with impatience for the operation, and refused every other treatment. The operation of trepan was resolved upon, in the situation where the pain was most severe. After making a crucial incision, the trephine was applied, and the osseous disc removed. The dura mater, and the internal surface of the vitreous table, were sound; nor did exudation exist between the two lamellæ of the bone. The periosteum, which was in part detached from the disc, was now entirely removed. The portion of bone was replaced into the opening, and the scalp over it retained in contact by means of adhesive plaster.

The febrile symptoms were moderate; the inflammation of the dura mater was by no means severe, and entirely local; but re-union of the flaps did not take place. Suppuration supervened. The discharge continued during some months; yet the patient found himself better, and the pain of the head gradually diminished, and ultimately disappeared. At the bottom of the wound, the osseous disc could be felt with the probe, free and moveable. The operator conceived, at the end of the third month, that it ought to be removed; but, after having seized it with the forceps, instead of bringing away the piece of bone in its entire thickness, a very thin, angular, and ragged portion, consisting only of part of the external table, was removed. The inferior surface of this portion was rough and unequal: one of its margins was round, the other pointed and serrated. In a word, the vitreous table of the separated disc and a part of the external lamella were re-united; while the larger portion of the latter was exfoliated. On attentive examination of the bottom of the wound by the probe, the parietal opening was found thoroughly closed, and filled by osseous matter, hard, and covered by healthy granulations.

As exfoliation and granulation cannot take place unless from a part which possesses an active state of its vessels, so it becomes evident that the re-united portion of bone retained its vitality in this case, formed vascular connexions with the dura mater and with the diploë, and became subject to the usual processes of nutrition and vascular action.

After the removal of the exfoliation, the suppuration gradually diminished, and in a short time the wound cicatrized in the usual manner. —*Nouveau Journale de Medecine.*

### 3. *On the Use of Sulphate of Quinine in Ague.*

A man, who had been twice before cured of ague, and once by the bark in substance, had a third attack of tertian fever, which commenced in August, and now resisted the bark and various other remedies. On the 29th of November, his countenance was of a pallid hue, as is usually seen after repeated attacks of this disorder. His strength and spirits were depressed; he had anasarcous swellings of the arms and legs, and ascites, with enlargement and induration of the spleen projecting below the ribs. The Doctor thought he recognized œdema of the lungs, by the great dyspnœa and cough, and especially by the sound transmitted through the stethoscope, which was obscure and deep, and observable over the whole surface of the lungs, or was accompanied by a noise similar to that which is produced in rubbing satin paper. Percussion gave a dull and doubtful sound.

An ounce of the bark in substance, given during the apyrexia, (we presume, regularly,) produced no change; when, on the 17th of December, on the evening preceding the expected paroxysm, the patient took twelve grains of sulphate of quinine, divided into six parts, one every two hours. On the 18th, the paroxysm did not make its appearance. On the 20th, he took six grains; on the 27th, four. Since the cessation of the fever, the secretion of urine increased; the spleen softened, and diminished in size; the respiration became improved; the appetite returned; and the aqueous deposits disappeared.

M. B., after several attacks of intermittent, which were cured by cinchona, had a return of fever, with violent cough and difficulty of breathing; the pulmonary symptoms terminating with the paroxysm. Cinchona was again tried, but its effects were not satisfactory. The writer then gave two grains of the sulphate of quinine every two hours, so that twelve were intended to be taken during the intermission. The paroxysm did not, however, return.

J. C., subject to attacks of intermittent, consulted Dr. Fallot on the last accession of fever, which was of a tertian type. Cinchona having failed to relieve, six grains of the sulphate of quinine were given on the 14th, and eight grains on the 15th. The paroxysm was retarded three hours on the 16th, and was shorter than usual. On the 18th, she took twelve grains in six doses; and on the 19th she had no paroxysms.—*Journale Complementaire,* May 1822.

### 4. *Excision of Bronchocele.*

The following is the case of Excision of Bronchocele, by Mr. Dupuytren, referred to in the Retrospect, (p. 45.)

A female applied at the Hôtel Dieu in Paris, for the removal of a large bronchocele, by which she had long been distressed. Her employment was rural, her temperament bilio-sanguineous, and constitution sound. Eight years previously, a tumor, large as a filbert, was perceived on the anterior and middle portion of her throat. During six years its growth was constant, but gradual and slow; in the seventh, it rapidly increased. Its progress now was rapid; it covered the whole front of the neck, and had three distinct lobes. The central one only was painful; it extended over the sternum, four inches in diameter.

A Parisian surgeon removed the diseased parts. No hemorrhage followed; and at the end of a month the wound cicatrized.

About six months afterwards, the two lateral lobes acquired an enormous size; the central also enlarged; deglutition was embarrassed, and the respiration rendered laborious and afflictive. Urged by these circumstances, and the unhappiness arising from a sense of deformity, she declared to the surgeons her fixed resolution of submitting to excision of the tumor, whatever should be the result. Drs. Pelletan and Dupuytren gave their explicit opinion that such an operation was altogether impracticable. Nevertheless, she returned in a few days, and intreated its performance. It was again declined; and the danger to which it would expose her described in the strongest terms. Palliatives were prescribed; but neither apprehension of pain nor of danger, nor formal refusal, could alter her purpose. Displeased and desperate, she appeared a third time, and pressed her original request. Such great perseverance at last excited a deep interest in her behalf; and she was admitted, on January 1, 1808, into the hospital, where the state of her neck was again minutely examined.

The anterior and lateral regions of the throat were occupied by a tumor, stretching perpendicularly from the inferior maxilla to the sternum and clavicles, and transversely from the one maxillary angle to that of the opposite side. It measured seven inches in length, and the same in breadth, and consisted of three soft and knobbed lobes. The central was least protuberant; it adhered to, and moved simultaneously with, the larynx. The lateral lobes were attached to the contiguous parts, and admitted motion in all directions. The integuments were flaccid; the jugular veins, and their ramifications, much dilated; the pulsations of the superior thyroid arteries, above the tumor, were violent and perceptible; the carotids throbbed with great activity and distinctness. Pain was not felt in the tumor; but it sensibly impeded respiration, obstructed deglutition, and, when the patient experienced lively emotions, produced irregularity of the cerebral circulation. Her face, on such occasions, assumed a deep red hue; and dazzling of the eyes, with vertigo, supervened.

The recent progress of this dreadful affection, with the sense of imminent suffocation experienced by the patient, formed certain indications of a fatal issue. On the other hand, the situation, size, and connexions of the tumor, displayed the danger of attempting its excision. After mature deliberation, therefore, it was once more pronounced impracticable. This declaration overwhelmed the unfortunate sufferer: she was seized with gloomy despair—with profound melancholy; she adopted the resolution of accelerating death by inanition, and firmly rejected all kinds of food. Her menstrual discharge, hitherto regular, now became suppressed. Violent spasms succeeded, with a fearful sense of suffocation, convulsions, horror, and excruciating pains.

Immediate dissolution, in these circumstances, was inevitable. By the operation, she might possibly be saved. It was promised; and her mind became calm and animated. Her sound constitution, strength, youth, and earnest desire for relief,—the great mobility of the tumor, its slight union with the integuments, and the knowledge of a partial extirpation of the parts having already been successfully made,—all presented themselves as motives of hope to M. Dupuytren, and determined him to undertake it. Accordingly, with the assistance of M. Rullier and others, it was performed, in the presence of Professor Pelletan and many surgeons of the French metropolis.

*Operation.*—The integuments of the anterior and middle parts of the neck being elevated, so as to form a transverse fold, they were divided perpendicularly in the centre down to its base. The incision was then prolonged upwards to the symphysis of the chin, and downwards to the superior border of the sternum. The skin of the left half of the tumor was now detached by careful dissection. In the course of this, two sets of veins were discovered; only lying close to the tumor, the other subcutaneous. Most of these were avoided; the rest were tied, and divided between the ligatures. On the left and posterior side of the tumor, four thyroid arteries, greatly enlarged, were easily recognized. Being denuded and doubly secured, they were divided between the two ligatures. In this manner the left half of the tumor was detached, with no other inconvenience to the patient than the pain induced by a protracted dissection.

The right lobe was then separated, with equal precaution. Its connexion with the circumjacent parts readily yielded to the finger and handle of the bistoury. Without difficulty, also, the internal jugular veins, carotid arteries, and pneumo-gastric nerves, were detected, and pushed aside from the way of the knife. Now M. Dupuytren ascertained the practicability of removing the whole mass. For this purpose the lateral portions of the tumor, which had been separated from their attachments, were turned up, and drawn forward so as to stretch the central lobe, which adhered closely to the larynx and trachea. The cellular substance, which connected it to these parts, was now divided with the knife, when the wind-pipe came into view: its surface was flattened by the pressure upon it by the incumbent tumor. Lint was placed in the bottom of the wound, its lips approximated, a light dressing superadded, and the ends of the ligatures left in its inferior angle.

The patient endured this tedious and intricate operation with fortitude. She lost only a few spoonfuls of blood. Once or twice she was threatened with syncope, and was sick. After the operation, her face was pale, its expression altered; her strength, in every respect, overpowered; the pulse was frequent, small, and concentrated; the respiration laborious and rapid; the skin cold. Cardialgia and constant nausea were established; and she appeared overpowered by the mere effect of the operation. To recruit and support her strength now became the prominent indication. Cordials were administered in small proportions. She was threatened with suffocation, on taking even a spoonful of liquid; and this could only be done by her when in a vertical posture. Towards the evening, however, her symptoms began to improve. Prostration of strength was now succeeded by re-action; the pulse was frequent and full; respiration nearly natural; the countenance flushed; the skin hot and dry; and some spoonfuls of fluid were introduced into her stomach, without inconvenience. But the hopes thus awakened were of short duration. By the evening of next day, her breathing became painful and stertorous; and she died.—*London Repository*, March 1822.

### 5. *Removal of Osteo-sarcoma of the lower Jaw.*

On the 17th November, 1821, Dr. Mott, of New York, operated upon a young lady, about seventeen years of age, with an osteo-sarcomatous affection of the lower jaw. The tumor involved the right side of the lower jaw, from about half an inch from the symphysis to beyond the angle, nearly as far up as the processes; occupied half the mouth; and extended to the avula, and downwards upon the neck, nearly to the thyroid cartilage. The carotid artery being previously tied, the first incision was made over the lower edge of the tumor, from a little above the lobe of the ear, in a semicircular direction by the side of the neck, and terminated on the chin near the corner of the mouth. The incisions with the saw were made on the fore-part of the bone, perpendicularly downwards, by the side of the first incisor tooth; and, in a transverse direction, about one-eighth of an inch below the semi-lunar line, which extends between the coronoid and condyloid processes. The wounds all healed kindly by the first intention, without the occurrence of a single unpleasant symptom; and the patient left the city for her home, perfectly well, on the 20th of December.—*American Medical Recorder*.

### METEOROLOGICAL JOURNAL.

By Messrs. WILLIAM HARRIS and Co. 50, Holborn, London.

*From May 20, 1822, to June 19, 1822.*

| | MOON. | Rain gauge | THERM. | | | BAROM. | | DE LUC'S HYG. | | WIND. | | ATMOSPHERIC VARIATION. | | |
|---|---|---|---|---|---|---|---|---|---|---|---|---|---|---|
| | | | 9 A.M. | MAX. | MIN. | 9 A.M. | 10P.M. | 9 A.M. | 10P.M. | 9 A.M. | 10P.M. | 9 A.M. | 2 P.M. | 10P.M. |
| May 20 | ● | | 63 | 71 | 51 | 30.05 | 30.06 | 58 | 60 | SSE | NE | Fine | | |
| 21 | | | 61 | 73 | 50 | 30.11 | 30.21 | 45 | 60 | E | SE | Fine | | |
| 22 | | | 55 | 68 | 51 | 30.28 | 30.09 | 55 | 63 | E var. | NW | Fine | | |
| 23 | | | 56 | 68 | 46 | 30.00 | 29.98 | 60 | 57 | E var. | E var. | Fine | | |
| 24 | | | 50 | 60 | 51 | 29.85 | 29.91 | 55 | 56 | E var. | ENE | Fine | | |
| 25 | | .10 | 56 | 64 | 56 | 29.90 | 29.81 | 59 | 62 | E var. | NW | Rain and | Th.&Lig | Fair |
| 26 | | .13 | 60 | 69 | 53 | 29.83 | 29.87 | 71 | 73 | NW | NW | Rain | | Cloud. |
| 27 | ☾ | | 57 | 64 | 57 | 29.90 | 30.12 | 65 | 61 | W | W | Fine | | Sho'ry |
| 28 | | | 61 | 66 | 60 | 30.01 | 30.14 | 54 | 57 | W | SW | Fine | | |
| 29 | | .05 | 65 | 70 | 59 | 30.07 | 30 08 | 50 | 50 | WSW | W | Fine | | |
| 30 | | | 64 | 68 | 57 | 30.11 | 30.17 | 51 | 55 | W | W | Fine | | |
| 31 | | | 64 | 71 | 61 | 30.21 | 30.17 | 53 | 48 | WNW | NW | Fine | | |
| June 1 | | | 67 | 73 | 60 | 30.11 | 30.14 | 42 | 51 | WNW | SE | Fine | | |
| 2 | | | 66 | 73 | 58 | 30.13 | 30.15 | 48 | 53 | SE | SSE | Fine | | |
| 3 | | | 70 | 77 | 63 | 30.15 | 30.11 | 53 | 45 | SSE | SE | Fine | | |
| 4 | ○ | | 71 | 81 | 64 | 30.13 | 30.11 | 48 | 48 | ENE | E var. | Fine | | |
| 5 | | | 70 | 79 | 64 | 30.08 | 30.10 | 37 | 47 | ESE | E var. | Fine | | |
| 6 | | | 71 | 80 | 60 | 30.11 | 30.12 | 41 | 45 | NE | ESE | Fine | | |
| 7 | | | 65 | 73 | 55 | 30.10 | 30.03 | 46 | 51 | ENE | SE | Fine | | |
| 8 | | | 63 | 77 | 61 | 29.96 | 29.89 | 56 | 57 | E var. | ESE | Fine | | Fair |
| 9 | | | 72 | 80 | 65 | 29.83 | 29.88 | 54 | 50 | ESE | SE | Fine | | |
| 10 | | .23 | 78 | 85 | 65 | 29.88 | 29.94 | 48 | 65 | E | SW | Fine | | Li.&R |
| 11 | | | 73 | 80 | 58 | 30.04 | 30.00 | 61 | 64 | ENE | E var. | Fair | Fine | |
| 12 | ☽ | | 61 | 71 | 54 | 30.05 | 30.00 | 50 | 55 | E | E | Fine | | |
| 13 | | | 60 | 69 | 57 | 30.00 | 29.99 | 45 | 48 | E | SE | Fine | | |
| 14 | | | 62 | 70 | 59 | 30.00 | 30.03 | 53 | 55 | ENE | E var. | Fine | | |
| 15 | | .51 | 61 | 69 | 59 | 30.00 | 30.06 | 60 | 63 | NW | SE | Fine | Storm | Rain |
| 16 | | | 62 | 73 | 60 | 30.15 | 30.11 | 60 | 61 | E var. | E var. | Fair | Fine | |
| 17 | | | 64 | 75 | 60 | 30.12 | 30.10 | 45 | 47 | ESE | ESE | Fine | | |
| 18 | | | 65 | 76 | 57 | 29.08 | 29.97 | 47 | 49 | SE | SE | Foggy | Fine | |
| 19 | ● | .05 | 60 | 71 | 52 | 29.90 | 29.83 | 50 | 51 | SE | SE | Fine | | |

The quantity of rain during the month of May, was 1 inch and 30-100ths.

